# Opinion: No

**DOI:** 10.1590/S1677-5538.IBJU.2016.04.03

**Published:** 2016

**Authors:** A. Lenore Ackerman, Shlomo Raz

**Affiliations:** 1 Department of Surgery, Division of Urology, Cedars-Sinai Medical Center, Los Angeles, CA, USA; 2 Department of Urology, David Geffen School of Medicine at UCLA, Los Angeles, CA, USA

**Keywords:** Suburethral Slings, Surgical Mesh, Pelvic Floor, Prolapse

Urinary incontinence is a highly prevalent condition affecting up to half of all women, most of whom have a component of stress urinary incontinence (SUI) (1). Approximately 10% of these women will undergo surgical treatment. While for decades the standard of care was the Burch colposuspension or autologous fascial pubovaginal sling, rapid advances in the development of augmented polypropylene products (APM) for medical use led to the widespread adoption of synthetic midurethral slings (SMUS) for the treatment of SUI.

SMUS are now the standard of care and the most commonly performed procedure for the treatment of SUI worldwide. Multiple level one randomized trials have demonstrated equivalent outcomes for SMUS to Burch colposuspension and autologous fascial slings (2). In addition to their ease of use, SMUS provide faster operative times, reduced post-operative morbidity, faster resumption of normal activity, and reduced cost (3). Patient satisfaction with the procedures has been quite high (4) with comparable durability to the older techniques.

As seen for slings, the adoption of APM products for the treatment of pelvic organ prolapse (POP) gained rapid acceptance due to perceived safety, efficacy, and durability. With increasing use, however, a growing number of severe complications came to light, prompting the FDA to release its first black box warning in 2008 (5). Such concerns continue to grow; after an expanded warning reissued in 2011 (6), the past year has seen the FDA upgrade of transvaginal mesh for the treatment of POP to a class III (“high-risk”) device (7).

Transvaginally-placed mesh used in the treatment of incontinence, however, remains exempt from these warnings, despite the fact that more than half of the tens of thousands of lawsuits brought against mesh manufacturers have been from patients implanted with retropubic or transobturator SMUS (8). Of the nearly one thousand patients seen at our center over the past five years for transvaginal mesh complications, 77% (n=747) were related to SMUS placement. The last FDA safety communication from 2011 notes that almost half of the Medical Device Reports (MDRs) for urogynecological meshes in the Manufacturer and User Device Experience (MAUDE) database were associated with SUI repairs, stating that the “FDA continues to evaluate the effects of using surgical mesh for the treatment of SUI and will report about that usage at a later date” (6).

All procedures have potential complications; any time we take scalpel to skin, the conceivable outcomes range from success to death. Risk denotes the possible range of negative outcomes following an intervention, while benefit details the potential positive outcomes to the patient. In contrast, an assessment of safety and efficacy require an understanding not just of the frequency with which these risks and benefits occur, but of their severity. Both of these are lacking for SMUS.

Multiple studies have detailed a wide range of complications after SMUS placement; this report is not meant to be an inclusive description of all the potential complications associated with SMUS placement, but instead seeks to highlight some of the limitations in the dialogue regarding SMUS complications. The common complications of clinical significance after SMUS placement include vaginal mesh extrusion, vaginal scarring/stenosis, urethral and bladder perforation, voiding dysfunction, de novo urgency and urge incontinence, recurrent infections, vaginal bleeding, dyspareunia, and chronic pain, which have been discussed in multiple comprehensive reviews (9) and studies (10). Instead, we will focus on a discussion of chronic pain as a representative example of this class of complications.

Chronic pain is probably one of the most neglected, yet prevalent complications after SMUS. Indeed, many studies only assessed pain at a single early time point in follow-up, frequently at 6 weeks after surgery. Those studies that did address chronic pain report incidences ranging from 0-31% (9); the highly variable methodologies and follow-up intervals make a comparison of these studies challenging. The Cochrane review reports an overall risk of chronic groin pain of 4.5% (2), but does not comprehensively address other sites of pain that can be affected after SMUS. It is also important to remember that the reported rates of success and adverse events frequently do not incorporate these types of complications at all. The continent patient with refractory, debilitating chronic pain developing more than one year after placement is typically considered a treatment success by multiple measures in the vast majority of clinical trials. The treatment of these patients is challenging and frustrating for both the patient and provider. No recommendations or treatment algorithms exist to guide treatment; so, many women will attempt myriad treatments before finally seeking partial or complete mesh excision. Pain is one of the most common indications for mesh removal, particularly for transobturator slings (11, 12). Even with excision, approximately one-quarter of these patients will not improve or will even worsen (13), living with constant, debilitating pain.

Compelling evidence (9, 14) suggests that the numbers of complications from SMUS are underreported, due to variability in outcome assessment methodology, reporting biases, and lack of long-term follow-up. Despite this underestimation, Blaivas et al. (9) estimate that at least one in ten (15.3%) women undergoing placement of a SMUS will experience a serious adverse event or surgical failure. A recent 5-year trial of women undergoing SMUS for SUI reported a much higher number of almost 25% (10), suggesting serious complications after SMUS are not rare.

More importantly, our understanding of the severity of complications and their impact on patient quality of life is drastically limited. A wide range of complications, such as recurrent cystitis, voiding dysfunction, de novo urgency and urge incontinence, neurologic symptoms, and chronic pain, are frequently trivialized in prospective studies as short-lived or controllable with expectant management. These studies, however, lack any long-term follow-up or quantitative assessment to support such dismissive claims. In a qualitative assessment of women with vaginal mesh complications, Dunn et al. (15) describe the degraded emotional and physical health of patients with these types of complications, particularly chronic pain. These women describe significant shame, hopelessness, regret, frustration, and anxiety impacting their personal relationships, self-image, and personal and professional productivity. In support of their findings, our anecdotal experience with over 1500 patients presenting with transvaginal mesh complications underscores the physical and psychosocial toll and the treatment-refractory nature of these complications on a subset of these patients.

The FDA states that “the safety and effectiveness of multi-incision slings is well-established in clinical trials that followed patients for up to 1 year” (6). Recent reports indicate that many of the refractory complications of SMUS do not present until years after placement. In a recent prospective trial (10), one-quarter of all women experienced significant adverse outcomes by five years after SMUS placement; almost 20% of all women who underwent SMUS were impacted by chronic pain. Multiple retrospective reviews reveal that the majority of these complications, especially pain, overwhelmingly develop more than a year after placement (11, 16). In our series of 747 women, 61% described refractory pain symptoms; of those, the average time to presentation was greater than three years after the initial surgery. Yet in the recent updated Cochrane review, only four of the 84 included trials addressed SMUS outcomes after one year (2), meaning that determinations regarding the safety of SMUS are being made using data with only months of follow-up, which may have limited clinical relevance.

The vast majority of commercially-available SMUS use polypropylene, a substance widely used in medical implants and surgical sutures with good tolerability (17). Despite this record, data increasingly suggest that there may be something unique to transvaginal surgery that alters the risk profile of mesh implants, resulting in greater risk of complications. The vagina cannot be completely sterilized by surgical preparation (18); thus, SMUS are placed through a contaminated field. While others have observed subclinical mesh infection in patients with chronic complications (19), our preliminary studies (20) have observed the enrichment in pathogenic bacterial species of SMUS in patients with chronic pain, while sling meshes removed for urinary retention lack this colonization. These patients also exhibit a macrophage-predominant peri-mesh inflammation that again is lacking from patients with urinary retention. This correlation implicates contamination of the SMUS surface during placement in the etiology of chronic pain and explains the unique complications after transvaginal surgery.

The serious complications of transvaginal POP mesh are well recognized. Logically, it is difficult to accept that a similar composition mesh implanted a few centimeters distally would be completely insusceptible to these devastating complications. It is more plausible that the increased rates of complications with POP meshes over those seen for SMUS may reflect the larger amount of mesh. Lighter weight meshes are better tolerated than the older thicker grafts (19); thus SMUS may be better tolerated than POP mesh simply due to the smaller volume of mesh used.

As physicians, we must come back to the principal precepts of bioethics: autonomy, justice, beneficence, and non-maleficence. Non-maleficence, often popularized by the phrase “first, do no harm”, purports it may be better not to do something if it risks causing more harm than good. Incontinence is a disease affecting quality of life; any treatment we prescribe should improve that quality. We must have the discussion as a discipline about whether even low incidences of severe, debilitating complications are reasonable for patients with a non-life-threatening illness, especially when equivalent, lower-risk alternatives exist.

The American Urogynecologic Society (AUGS) and the Society for Urodynamics, Female Pelvic medicine and Urogenital Reconstruction (SUFU), published a joint position statement supporting the continued use of SMUS for the treatment of SUI. This report does not intend to challenge those conclusions. We do not pursue a ban on the use of synthetic mesh for incontinence surgery; millions of patients have benefitted tremendously from its use. We seek only to highlight the limitations of our current knowledge and expand the debate regarding mesh-augmented procedures in SUI management. Even with conservative estimates, several percent of patients receiving SMUS may have devastating, irreversible complications that drastically alter their lives. We must consider whether the improvements in perioperative morbidity and recovery for SMUS over the older Burch colposuspension and autologous fascial sling procedures are justifiable in the face of these serious adverse events. If the answer is affirmative, our shaping of pre-operative expectations must reflect the seriousness of these possible poor outcomes.

While slings have been extensively studied over decades, we may not have been asking several vital questions defining long-term satisfaction, success, and adverse events. There is not as yet adequate data to address this knowledge gap; we must perform long-term prospective studies to define the nature and severity of the range of complications following SMUS and determine how these newer techniques measure up against historical standards. Without complete data, we have allowed the scientific debate to be determined by the legal system, devolving medical practice into litigious mudslinging.

Perceived treatment failure on the part of the patient has less to do with objective or even subjective symptomatic improvement and more to do with the patient's expectations of outcomes. If we frame SMUS surgery as a quick fix for everyone with few side effects, we may continue to see a growing divide between physicians and patients and continue to contribute to the emotional and psychological devastation these patients experience.

It is hard to remember that there is a low risk of debilitating pelvic pain when you are the one affected. While slings may be effective for most patients, the past decade has seen the evolution of high-volume tertiary referral centers specializing in the treatment of mesh complications. Patients have self-organized into advocacy and support groups and have even created their own registries of complications; all of which suggests that the medical profession has failed to adequately address and acknowledge these patients' experiences. As a community we must take this public outcry seriously, acknowledge our lack of insight into these complications, and pursue a deeper understanding of the pathophysiology of poor outcomes after SMUS.

Like all new technologies, the SMUS is not perfect. But it has been beneficial to large numbers of women, restoring their ability to function in society without incontinence. The recognition, comprehensive characterization, and deeper understanding of the many complications after SMUS could lead to the development of better, lower risk implants with better durability for all patients. We owe it to them.

## ABBREVIATIONS

APM: = Augmented Polypropylene Mesh
AUGS: = American Urogynecologic Society
FDA: = Food and Drug Administration
MDR: = Medical Device Reports
MAUDE: = Manufacturer and User Device Experience
POP: = Pelvic Organ Prolapse
SMUS: = Synthetic Mid-Urethral Sling
SUFU: = Society for Urodynamics, Female Pelvic Medicine and Urogenital Reconstruction
SUI: = Stress Urinary Incontinence
